# Sex Differences in Mouse Cardiac Electrophysiology Revealed by Simultaneous Imaging of Excitation-Contraction Coupling

**DOI:** 10.3390/jcdd10120479

**Published:** 2023-11-29

**Authors:** James I. Emerson, Pablo Ariel, Wei Shi, Frank L. Conlon

**Affiliations:** 1 Department of Biochemistry and Biophysics, University of North Carolina at Chapel Hill, Chapel Hill, NC 27599, USA; james_emerson@unc.edu; 2 Microscopy Services Laboratory, Department of Pathology and Laboratory Medicine, University of North Carolina at Chapel Hill, Chapel Hill, NC 27599, USA; pablo_ariel@med.unc.edu; 3 Department of Biology and Genetics, McAllister Heart Institute, University of North Carolina at Chapel Hill, Chapel Hill, NC 27599, USA; shiw618@email.unc.edu; 4 Integrative Program for Biological and Genome Sciences, University of North Carolina at Chapel Hill, Chapel Hill, NC 27599, USA

**Keywords:** sex differences, cardiac electrophysiology, excitation-contraction coupling

## Abstract

Males and females differ in the basic anatomy and physiology of the heart. Sex differences are evident in cardiac repolarization in humans; women have longer corrected QT and JT intervals. However, the molecular mechanisms that lead to these differences are incompletely understood. Here, we present that, like in humans, sex differences in QT and JT intervals exist in mouse models; female mice had longer corrected QT and JT intervals compared with age-matched males. To further understand the molecular underpinning of these sex differences, we developed a novel technology using fluorescent confocal microscopy that allows the simultaneous visualization of action potential, Ca^2+^ transients, and contractions in isolated cardiomyocytes at a high temporal resolution. From this approach, we uncovered that females at baseline have increased action potential duration, decreased Ca^2+^ release and reuptake rates, and decreased contraction and relaxation velocities compared with males. Additionally, males had a shorter overall time from action potential onset to peak contraction. In aggregate, our studies uncovered male and female differences in excitation-contraction coupling that account for differences observed in the EKG. Overall, a better understanding of sex differences in electrophysiology is essential for equitably treating cardiac disease.

## 1. Introduction

Various human disease states exhibit sex differences in prevalence, treatment, and survival, including cancer, cardiovascular disease, autoimmune disorders, obesity, and chronic kidney disease [1,2,3,4,5]. Sex disparities are evident in the function and pathologies of the heart. Cardiac arrhythmias are diseases with diverse etiology that exhibit profound sex differences in prevalence [1,6,7]. Women account for up to 70% of arrhythmia diseases such as long QT syndrome (LQTS), and diseases such as atrial fibrillation are more common in men [8,9,10,11]. Arrhythmias occur due to defects in the heart’s conduction system. The cardiac conduction system provides a low-resistance pathway for action potentials to stimulate synchronous contraction across cardiomyocytes in a process termed excitation-contraction coupling. Human sex differences in this pathway are evident because women have an elevated resting heart rate, longer cardiac repolarization, and smaller cardiac output compared with men [12,13,14,15,16]. These physiological sex differences can predispose to developing arrhythmias.

Since the advent of the electrocardiogram (EKG), investigators have observed that women display, on average, 20 ms longer times from ventricular depolarization to repolarization, corrected QT (QTc) intervals, compared with men [16,17,18,19]. Generally, for every 10 ms increase in QTc interval, there is a 5% increase in the risk of arrhythmic events [20]. This baseline sex difference predisposes women to develop LQTS [12,21,22]. Therefore, understanding the molecular mechanisms that lead to electrophysiological sex differences is vital for treating LQTS and other sex-differential arrhythmias. Sex differences in repolarization are conserved in most vertebrate species [12,22,23]; however, studies of sex differences in QTc interval in mice have produced conflicting results [24,25,26,27,28,29,30,31]. Trepanier-Boulay et al. and Rodgers et al. reported increased QTc interval in female mice from both the C57 and CD1 background [24,27], whereas Brunet et al. reported an absence of differences [25]. Warhol et al. found QTc differences only in mice under anesthesia [28], and Saito et al. found QTc differences only while female mice were in estrus [31]. Therefore, more studies with greater statistical power are necessary to better understand mouse repolarization differences.

Increased repolarization time is generally paralleled at the molecular level by prolonged action potential duration (APD) and calcium (Ca^2+^) reuptake times in female rabbits, dogs, and guinea pigs [12,23]. However, contradictions are noted in measuring APD of isolated mouse cardiomyocytes [24,25,26,31]. Sex differences in APD are reported in cells of the interventricular septum and in mice in estrus. These differences are attributed to hormonal influence and decreased expression of repolarizing K^+^ channels in females [24,25,26]. Reporting of Ca^2+^ parameters is more consistent; decreased rates of Ca^2+^ release and reuptake in female animals is reported across species [32,33,34,35]. Studies of contraction of isolated cardiomyocytes (CMs) from mice and rats have shown increased rates of contraction, relaxation, and overall percent contraction in males [32,34,36,37,38]. Sex differences in contraction and Ca^2+^ transients are attributed separately to basal sex differences in cyclic adenosine monophosphate (cAMP) levels [33,39] and insulin-like growth factor (IGF-1) deficiency [37]. Overall, sex differences exist in each step of excitation-contraction coupling, highlighting the importance of this mechanism in sex-differential physiology and disease.

Despite the precise characterization of individual excitation-contraction coupling events, there are limited tools able to integrate action potential, Ca^2+^ transient, and contraction on the same time base. Techniques such as patch clamping or optical imaging have enabled precise detection of any one or a combination of these parameters [33,39,40,41,42]. van Meer et al. and Nguyen et al. reported simultaneous optical imaging using induced pluripotent stem cell-derived cardiomyocytes [43,44]; however, simultaneous imaging has not been achieved with primary cells isolated from the myocardium. Isolated CMs provide valuable physiological understanding, and new methods have improved the efficiency and accessibility of CM isolation [45]. Given that membrane depolarization, Ca^2+^ release, and contraction are highly connected and tightly regulated, there is a critical need to simultaneously record these parameters in isolated CMs to further understanding of electrophysiology.

Here, we present an integrated method to simultaneously detect membrane potential changes, intracellular Ca^2+^ handling, and contraction from isolated CMs. Using fluorescent Ca^2+^ and membrane potential indicators with close to 1 kHz temporal resolution, we defined established and novel excitation-contraction coupling parameters. This procedure shortened the time between cardiomyocyte isolation and data acquisition to less than 3 h and enabled high-efficiency imaging of large cell populations. Using these novel methods, we have characterized the physiological sex differences between male and female mice. Our studies help resolve previous conflicting results in mouse EKGs by using a sample size with sufficient power to detect male–female differences across multiple time points. From EKG recordings, we report baseline sex differences in cardiac repolarization as indicated by QTc and JTc intervals. Consistent with this finding, action potential duration was increased in isolated female CMs. Like previous studies, we found that female mice displayed longer Ca^2+^ release and reuptake times compared with male mice. Contraction velocity and time were also reduced in males compared with females. We report sex differences in novel parameters, indicating baseline sex differences in coupling among action potential, Ca^2+^ release, and contraction. Overall, a better understanding of baseline sex differences in electrophysiology offers new and improved avenues for the treatment of sex-biased cardiac diseases.

## 2. Materials and Methods

### 2.1. Mice

Wild-type C57Bl6/J mice were obtained from Jackson Laboratories (Cat No. 000664). Mice were housed at controlled temperatures of 25 ± 1 °C, with a 12-h light/12-h dark cycle, with lights on from 07:00 to 19:00. Standard rodent chow and water were provided throughout the study period. The Institutional Animal Care and Use Committee at UNC-CH approved these mouse studies, which were performed in accordance with the Guide for the Care and Use of Laboratory Animals 22-257.

### 2.2. Mouse Surface Electrocardiography

EKG analysis of 4-, 6-, and 8-week-old male and female mice was performed at the UNC biomolecular research and imaging core facility as described [46,47]. Mouse EKGs were taken under live restraint (non-anesthetized). EKGs were recorded using Vevo 2100 ultrasound machine’s physiological acquisition mode, which provided voltage readings at 8000 Hz. The front two paws and a right rear paw were placed in contact with electrodes lightly coated with Sigma electrode creme (Parker Laboratories Inc., Fairfield, NJ, USA). Physiological data were exported from the Vevo 2100 machine and processed into .txt files, which enabled compatibility with Labchart software. EKGs were analyzed using the LabChart (v8.1.16) viewer mode application, whereby at least four consecutive waveforms were averaged to obtain EKG parameters for each mouse. Standard detection parameters were used for mouse EKGs, including typical QRS width of 10 ms and R waves at least 60 ms apart, measured ST height at 10 ms from alignment, and special detection of rodent t waves. Bazett’s correction was used to calculate QTc intervals.

### 2.3. Cardiomyocyte Isolation and Fluorescent Dyes

Cardiomyocytes (CMs) were isolated from adult C57 mice in a manner similar to described [45,48]. Briefly, mice were sacrificed by cervical dislocation, and the chest cavity was quickly opened. After cutting the descending aorta, 7 mL of EDTA buffer (all buffer compositions in supplementary materials) was quickly injected into the right ventricle. Next, a surgical string was used to tie a knot above the atria around the great vessels exiting the heart (vena cava and aorta). The heart was excised from the animal and placed in a dish containing 10 mL EDTA buffer. A syringe loaded with 10 mL EDTA buffer was used to inject into the left ventricle of the mouse heart. Tying of the great arteries was intended to create a perfusion flow wherein the left ventricle fed the coronary arteries, which perfused the rest of the heart with blood exiting from the hole made in the right ventricle. This perfusion was performed at a minimal flow rate (~2 mL per minute) to keep the heart distended. Next, the heart was moved into a dish containing 10 mL of perfusion buffer and injected with 3 mL of perfusion buffer into the same hole in the left ventricle. Next, the heart was transferred into a dish with 10 mL collagenase solution. The heart was perfused 3 times with 10 mL fresh collagenase solution, alternating perfusions between the left and right ventricle. At the conclusion of collagenase injections, the heart should appear light brown in color and be very soft. Next, the left ventricle (LV) and Interventricular septum (IVS) were isolated by removing both the atria and the right ventricular wall. LV and IVS tissues were transferred to a dish containing 3 mL collagenase buffer, and tweezers were used to physically dissociate the heart tissue. Once the tissue was sufficiently small (<5 mm), a 1 mL micropipette was used to gently agitate the tissue for 2 min, after which, 5 mL of stop buffer was added and pipetted gently for 2 min. To separate isolated CMs, the solution was passed over a 100 μm nylon filter into a 50-mL conical tube, followed by 5 mL of stop buffer to wash the plate and filter. The 50-mL tube with CMs was placed at a 45° angle and allowed to settle by gravity for 20 min (45° angle allows more surface area for CMs to spread out so they do not crowd each other and become ischemic). After the supernatant was removed, CMs were resuspended in 2 mL Ca^2+^ reintroduction buffer I. This process was repeated with Ca^2+^ reintroduction buffers II and III. After setting in Ca^2+^ reintroduction III, the CMs were placed in imaging media without BDM to add fluorescent dyes.

Fluorescent dyes were used to detect action potential and Ca^2+^ flux [49,50]. Fluo-volt (ThermoFisher cat# F10488) was used to detect action potentials, as used previously in mice, and induced pluripotent stem cell-derived cardiomyocytes [51,52]. Loading solution (10 μL) was mixed with 1 μL of dye separately and then added to a 1 mL solution of cardiomyocytes in imaging media. Intracellular Ca^2+^ dye Calbryte 590 (AAT Bioquest cat #20700) was added (5 μL per 1 mL) to the solution of CMs [53]. Cardiomyocytes were incubated for 15 min with both dyes. Afterward, the supernatant was removed, and cells were resuspended in fresh RT imaging media.

### 2.4. Simultaneous Imaging of Ca^2+^ Handling, Membrane Potential, and Contraction

After cells were prepared, they were transported in a 15-mL Falcon tube in an insulated box to the microscope. Transit time was less than 5 min. Experiments in the microscope room were performed at ambient temperature (23 °C).

Cells were transferred with a Pasteur pipette to a #1.5 coverslip in an IonOptix MyoPacer Field Stimulator with FHD microscope chamber system. This chamber was placed on a Zeiss LSM900 laser scanning confocal microscope, equipped with a Plan Apochromat 40X/1.4 oil immersion objective, 3 GaAsP photomultiplier tube (PMT) detectors for fluorescence, and one multialkali PMT for transmitted light detection. Once the stimulation chamber was placed on the microscope, stimulation was started with 1 Hz, 5 V intensity using bipolar waveforms lasting 4 ms, and left on continuously until the end of all imaging runs with the sample. Focus was found by eye, using the green fluorescence channel (Lumen Dynamics, X-Cite Series 120 Q light source; Zeiss filter set 10—excitation: 450–490 nm, dichroic: 510 nm, emission: 515–565 nm). Once cells were in focus, a systematic search was run in a snake pattern from the top left to the bottom right of the coverslip. Cells were selected for imaging if they fulfilled the following conditions, as assessed by eye: rod-shaped, contracting at 1 Hz, visible voltage flashes in the green fluorescence channel, not touching any neighboring cells on the sides, below or above. In a typical preparation, more than half of the rod-shaped cells surveyed fulfilled the selection criteria. The search process was fast, and cells were found and imaged at a rate of one a minute. Typically, 15–20 cells were imaged from a single tube of cells in 20–25 min, including set up time.

Imaging settings were loaded from a prior image, using the “Reuse” option in the software that controlled the microscope (Zen, blue edition, 3.5.093.00009). Three channels were imaged in line-mode, with line switching between different fluorescent channels, to generate an XT kymograph. Only isolated cells (no piles of overlapping cells) were imaged; therefore, optical sectioning was not needed, and the pinhole was opened fully to maximize sensitivity. For each line in the kymograph, a red fluorescence channel was acquired simultaneously with a transmitted light channel, after which a green fluorescence channel was acquired. This process was repeated for the entire kymograph. The red fluorescence channel used a 561 nm laser for excitation and collected light from the 576–700 nm range on a GaAsP PMT detector. Transmitted light was detected on a multialkali PMT. Excitation intensity on the 561 nm laser was set to 0.1%, which corresponded to approximately 5 μW at the specimen plane, measured with a Thorlabs S170C microscope slide power sensor and PM100D power meter. The green fluorescence channel used a 488 nm laser for excitation and collected light from the 400–576 nm range on a different GaAsP PMT detector. Excitation intensity on the 488 nm laser was set to 1%, which corresponded to approximately 50 μW at the specimen plane. The zoom was set to 0.45X and the live mode was used to look at the field of view briefly in the green fluorescence channel. The “line select” mode was used to draw a line through the long axis of the desired cell, parallel to its myofilaments, and always leaving some empty space on either side. The scan speed was set to 9, which corresponded to a line time for the three channels of 1.24 ms (imaging at 806 Hz). The line used for scanning always contained 512 pixels but could span slightly different lengths (range 0.2 to 0.35 μm); hence, the pixel size in μm could vary between cells, as could the power density of the laser on the sample. Five thousand lines were scanned, corresponding to a kymograph duration of 6.2 s. In a small fraction of imaging runs, the red fluorophore showed localized sparks or slow waves; those runs were discarded. All other imaging runs were saved as .czi files. These files were converted to an XT kymograph image (also in .czi format), using a version of Zen 3.0 (blue edition, version number 3.0.79) on another computer, by navigating to the XZ/XT tab and clicking on the “Generate X-Y image” option.

### 2.5. Image Analysis in ImageJ and Microsoft Excel

Before image analysis, data were organized in a specific folder structure that was necessary for custom-written macros to run correctly. In general, an experiment had at least two conditions (example: male, female), each of which included data from multiple animals. Approximately 15–20 imaging runs were performed on samples from each animal. Each imaging run was a kymograph from a single cell. Data from each animal (or combination animal + condition) were placed in a separate folder.

Once data were organized properly, a custom FIJI macro called MontageMakerFolders (see Supplemental Files) was run on the parent folder that contained multiple subfolders (one subfolder for each animal). This macro created a folder with one montage image per animal. Each montage image included all the kymographs from that animal, placed side by side. For each montage, a horizontal line was drawn across this image in the third channel (green fluorescence, voltage reporter). The fluorescence intensity profile along this line was plotted, and a threshold value was selected that would distinguish cells from the adjacent background. This value was between the background values outside the cell and the signal inside the cells.

Once a proper threshold value was selected, a custom FIJI macro called TraceCreator (see Supplemental Files) was run on each animal subfolder. For each kymograph, this macro extracted average fluorescence over time for the Ca^2+^ reporter and the voltage reporter channels, estimated the width of the cardiac cell over time, and corrected the Ca^2+^ and voltage reporter channel data for changes in cell width. The width of the cell was calculated based on a mask created on a median-filtered version of the voltage reporter channel, using the threshold value described above. The correction for cell width ensured that the average fluorescence at every time point was calculated only across pixels that were inside the cell, avoiding systematic errors due to changes in cell width. The macro saved images of the cell masks, masked versions of the original image data used for the width correction, and a .csv file with the measurements. Only the data corrected for cell width were used for further analysis.

The width and corrected fluorescence data were pasted into a custom Microsoft Excel (Microsoft 365 MSO version 2308) spreadsheet, called TracesAnalyzer (see Supplemental Files). This spreadsheet facilitated analysis of Ca^2+^, voltage, and width traces by providing interactive graphs, semiautomatic detection of inflection points where signals begin to rise, automatic detection of peaks and calculation of variables of interest, including: Ca^2+^ DF/F0, Ca^2+^ rise time, Ca^2+^ Tau1/2, Ca^2+^ Tau90, voltage DF/F0, voltage rise time, voltage Tau90, time delay between voltage and Ca^2+^ rise, temporal offset between Ca^2+^ and voltage peaks, changes in baseline after the first stimulus for Ca^2+^ and voltage, % change in width of cell, contraction time, Tau1/2 of cell contraction, Tau1/2 of cell relaxation, contraction speed, relaxation speed, temporal offset between voltage peak and contraction minimum, and temporal offset between Ca^2+^ peak and contraction minimum. A few parameters were adjusted manually using this spreadsheet to analyze traces from each imaging run. First, the start of a search window for detection of the first peak was set in each imaging run because we did not have a way to synchronize the electrical stimulation of the cells to specific frames in the imaging. Second, we included parameters to refine the automatic detection of when rises in Ca^2+^ and voltage signals began; sometimes the automatic estimates needed to be shifted manually by a few timepoints. Finally, our measurements of cell width could be quite noisy, so we included a parameter to vary the number of points used to fit a line to calculate the contraction and relaxation speed.

Once each imaging run was analyzed using the Excel spreadsheet, a specific section of the output was copied to another Excel worksheet called AnalysisSummarizer (see Supplemental Files). Pasting data from each imaging run into that worksheet enabled quick calculation of summary graphs and statistics.

All of the macros, worksheets, instructions for use, detailed explanations of calculations, and a sample data set are provided as supplemental data to this manuscript. https://cdr.lib.unc.edu/collections/k3569f699 (accessed on 11 November 2023).

### 2.6. Statistical Methods

Male–female differences in excitation-contraction coupling were determined using the Mann–Whitney ranked sum test with the Prism 9 (version 9.5.1) software. Two-factor ANOVA with Tukey’s multiple comparisons test was used to analyze differences with respect to sex and mouse age. All data are reported as mean ± standard error of the mean.

## 3. Results

### 3.1. Adult Mice Demonstrate Baseline Sex Differences in Cardiac Repolarization

**Figure 1:**
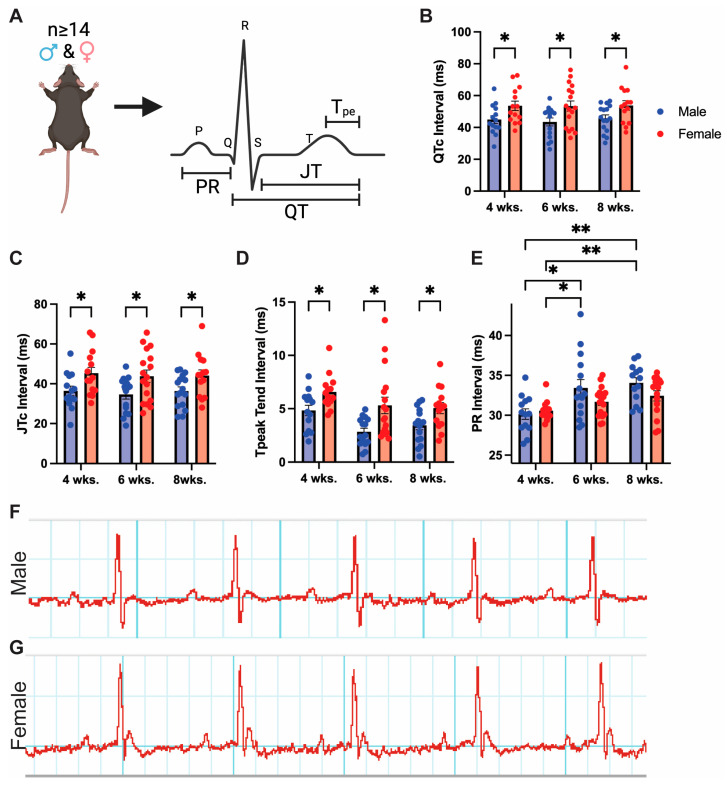
Sex differences in mouse electrocardiography. (**A**) Schematic of mouse EKG recording with key intervals indicated Male and female mice n ≥ 15 EKGs obtained at 4 and 6 weeks analyzed for (**B**) QTc, (**C**) JTc, (**D**) Tpeak to Tend interval, and (**E**) PR interval (Female data in red, male in blue). Representative (**F**) male and (**G**) female EKG trace taken from 6-week-old mice. * indicates *p* < 0.05, ** indicates *p* < 0.01 in ANOVA using Tukey’s multiple comparisons test.

It is well-established that in humans, females exhibit increased times for ventricular repolarization, as indicated by longer QTc and JT intervals [16,17,18,19,54,55,56]. Sex differences in repolarization are conserved throughout multiple vertebrate species, but previous results in C57BL/6 mice, the most widely used inbred strain of mice, are conflicting [25,27,28,31]. To determine if human sex differences are conserved in mice, we conducted surface electrocardiogram (EKG) recordings of adult male and female C57BL/6J mice aged 4, 6, and 8 weeks. Using power analysis, we determined that n ≥ 14 mice would be necessary to uncover if mice have differences in QTc, assuming QTc differs by 5–10% as in humans (Figure 1A, Supplemental Figure S1).

Female mice exhibited significantly longer QTc compared to age-matched males. A ~20% QTc increase in females was observed at 4, 6, and 8 weeks (Figure 1B). Moreover, female versus male mice display a significant 25% increase in JT intervals on average across all time points, indicating females experience longer times for ventricular repolarization than males (Figure 1C). JT interval differs from standard QTc interval as it does not consider the QRS complex, offering a better metric of repolarization time alone [57]. T_peak_ to T_end_ time measures the dispersion of repolarization across ventricular myocytes and can indicate the potential for arrhythmias [58]. We find that T_peak_ to T_end_ time is also increased across time points in females, indicating a greater propensity for the development of arrhythmias in female versus male mice at baseline (Figure 1D). The PR interval did not show significant sex differences from 4 to 8 weeks, though it trended to be longer in males at 6 and 8 weeks. This was due to a significant increase in male PR interval between 4 to 6 weeks, likely corresponding with the enlargement of atria over this period (Figure 1E). Female mice develop significantly longer QRS intervals as they age from 4 to 6 weeks, while ST height is significantly decreased over the same period (Supplementary Figure S2). Male QRS and ST height experienced the same trends, though they were non-significant. These changes correlate with increased ventricular growth over this time, which increases ventricular depolarization time. S amplitude was significantly larger in male myocytes at 4 and 8 weeks. Representing the depolarization of Purkinje fibers, this increased S wave amplitude in males may correspond to a larger overall heart mass, as reported previously [59]. Overall, female mice have an increased time for ventricular repolarization and greater dispersion of repolarization among the ventricles than males (Figure 1F,G). Therefore, our findings suggest that female C57BL/6J mice at baseline recapitulate repolarization differences observed in humans.

### 3.2. Simultaneous Detection of Cardiac Action Potential, Ca^2+^ Transient, and Contraction

**Figure 2:**
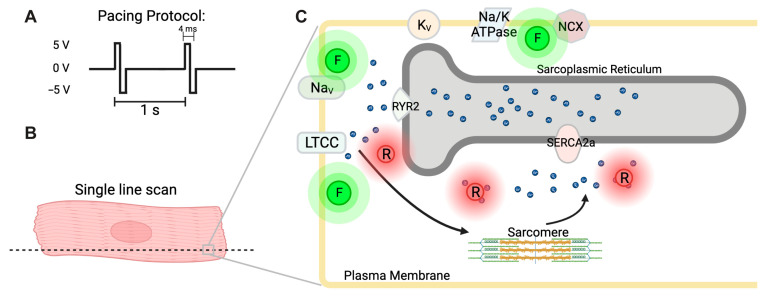
Graphical abstract of the simultaneous imaging method. (**A**) Pacing protocol schematic. (**B**) Imaging CMs using single-line scans using dual fluorescence. (**C**) Schematic of Excitation contraction coupling with key proteins and localization of fluorescent dyes used to detect membrane potential and cytoplasmic Ca^2+^ concentration. *F* fluovolt, *R* Cal-bryte 590, *NaV* voltage gated sodium channels, *LTCC* L-type Ca^2+^ channel, *KV* voltage gated potassium channel, *Na/K ATPase* Sodium-potassium pump, *NCX* Sodium-calcium exchanger, *RYR2* Ryanodine receptor 2, *SERCA2a* Sarco/endoplasmic reticulum ATPase 2, *small blue dots* Ca^2+^.

Knowing that repolarization differs in the surface EKG, we sought to develop a tool that would detect the underlying molecular events that govern cardiac electrophysiology. Excitation contraction coupling is a process in CMs by which action potentials lead to calcium-induced calcium release from the sarcoplasmic reticulum (SR) and subsequent contraction by Ca^2+^ binding and releasing inhibition of myofilaments. Simultaneous imaging and quantification of the three events in excitation-contraction coupling have not previously been reported in isolated CMs. To close this gap, we developed a dual fluorescence imaging system to record action potential, Ca^2+^ transient, and cell contraction simultaneously. Myocytes were paced using 5-volt bipolar pulses at 1 Hz frequency to stimulate excitation-contraction coupling (Figure 2A). Using dual fluorescent confocal microscopy, we were able to attain single-line scan images across the long axis of cardiomyocytes, enabling high imaging speeds near 1 kHz (Figure 2B). Fluo-volt dye was used to detect membrane potential changes by increased green fluorescence in response to more positive intracellular membrane potential (Figure 2C). Calbryte-590 detected intracellular Ca^2+^ concentration by increasing red fluorescence in response to binding Ca^2+^ in the cytoplasm (Figure 2C). We determined cell contraction parameters by thresholding of the kymographs obtained from this scheme. Overall, this method enabled unparalleled temporal resolution and quantification of excitation-contraction coupling.

### 3.3. Isolated Female Cardiomyocytes Exhibit Increased Action Potential Duration

**Figure 3:**
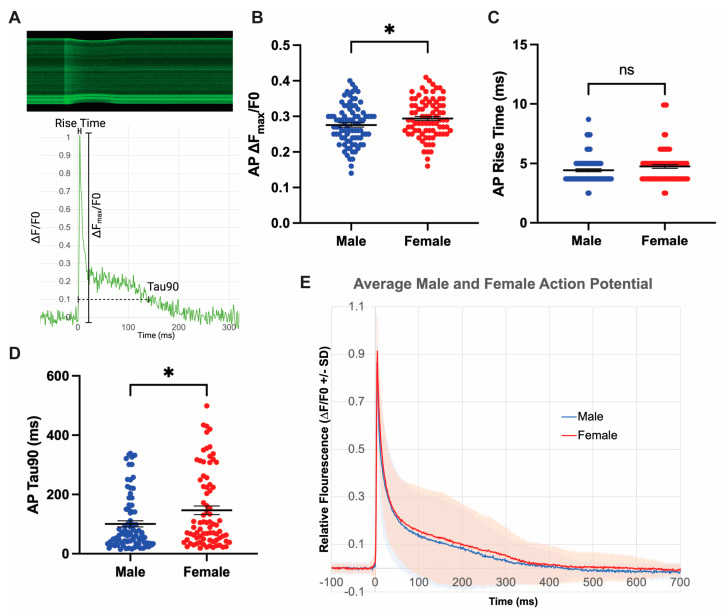
Isolated Female Cardiomyocytes exhibit Increased Action Potential Duration. (**A**) Kymograph of membrane potential, indicated by Fluo-volt, over 500 ms corresponding with representative action potential trace extracted from the kymograph. Key parameters are defined: Fluo-volt ∆F_max_/F0, repolarization time (Tau90), and Depolarization time (Rise time). Representative trace from a female cell with the following parameters: ∆F_max_/F0 0.22, rise time 3.72 ms, Tau90 143.84 ms. (**B**) Relative peak depolarization between males and females. (**C**) AP rise time in males and females. (**D**) Repolarization time in males and females. (**E**) Composite graph depicting average AP trace from males and females across all samples with standard deviation. Data presented from 5 male and 5 female mice, 84 male cells and 86 female cells. ns indicates *p* > 0.05, * indicates *p* < 0.05 in Mann–Whitney test.

The EKG represents the sum of membrane potential changes in individual cardiomyocytes. Therefore, alterations in single-cell action potential (AP) duration can lead to changes in QTc and JT intervals [12]. We employed fluorescent microscopy with Fluo-volt dye to quantify relative membrane potential in isolated left ventricular CMs from 6-week-old C57BL/6J mice (Figure 2). This method allowed quantification of Fluo-volt ∆F_max_/F0, AP rise time, and AP decay time (Figure 3A). We found that peak depolarization relative to baseline was greater in female myocytes (6%) compared to males (Figure 3B), though the relative difference was small. Both sexes had equivalent rise times from baseline to peak (Figure 3C), indicating similar rates of depolarization by sodium influx. Interestingly, females exhibit a significant 37% increase in APD90 times compared to males (Figure 3D,E). This finding corresponds with longer repolarization times observed in JT intervals between male and female mice (Supplemental Figure S3). Thus, female mice at baseline have increased action potential duration, which accounts for the longer repolarization times measured by the EKG.

### 3.4. Increased Rates of Ca^2+^ Release and Reuptake in Male Cardiomyocytes

**Figure 4:**
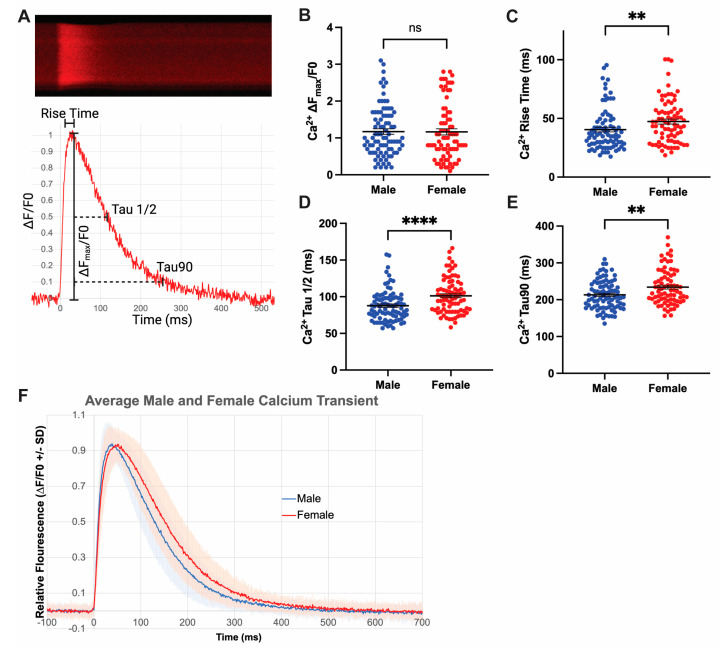
Increased rates of Ca^2+^ release and reuptake in male cardiomyocytes. (**A**) Kymograph of Ca^2+^ transient by Calbryte-590 over 500 ms corresponding with representative trace extracted from the kymograph. Key parameters are defined: Calbryte-590 ∆F_max_/F0, repolarization time at half (Tau ½) and 90% (Tau90), and time from baseline to peak release (Rise time). Representative trace from a female cell with the following parameters: ∆F_max_/F0 1.08, rise time 27.3 ms, Tau1/2 90.5 ms, Tau90 235.6 ms. (**B**) Relative peak Ca^2+^ release between males and females. (**C**) Ca^2+^ transient rise time in males and females. Ca^2+^ transient reuptake time in males and females at (**D**) 50% and (**E**) 90% reuptake. (**F**) Composite graph depicting average Ca^2+^ transient from males and females across all samples with standard deviation. Data presented from n = 5 mice, 84 male cells and 86 female cells. ns indicates *p* > 0.05, ** indicates *p* < 0.01, **** indicates *p* < 0.0001 in Mann–Whitney test.

Rapid CM depolarization triggers Ca^2+^ influx through L-type Ca^2+^ channels, which leads to calcium-induced calcium release from the SR [60]. Ca^2+^ dynamics are tightly regulated in the cell due to their critical importance in influencing contraction and action potential duration [60,61]. We visualized Ca^2+^ transients using the Calbryte-590 fluorescent Ca^2+^ indicator [53] (Figure 2), allowing quantification of Calbryte-590 ∆F_max_/F0 as well as transient rise and decay times (Figure 4A). Males and females exhibit similar ∆F_max_/F0, indicating similar peak cytoplasmic Ca^2+^ concentration relative to baseline (Figure 4B). Despite achieving the same peak concentration, male myocytes have faster rates of Ca^2+^ transient rise (Figure 4C). This suggests males have increased rates of Ca^2+^ release from the ryanodine receptor (RYR2) relative to females. Male myocytes also displayed faster Ca^2+^ transient decays to 50% and 90% of the peak, consistent with increased rates of Ca^2+^ reuptake back into the SR (Figure 4D,E). Overall, females versus males display a 10–20% decrease in rates of Ca^2+^ release and reuptake, while a similar peak Ca^2+^ concentration is achieved in females and males (Figure 4F). Given total peak cytoplasmic Ca^2+^ concentration in mice is primarily accounted for by Ca^2+^ release from the SR [62], differences observed in Ca^2+^ transients are most likely due to changes in SR Ca^2+^ release and reuptake. Decreased SR Ca^2+^ reuptake rates in females may also contribute to prolonged AP duration due to increased Ca^2+^ extrusion through the Na^+^/Ca^2+^ exchanger (NCX), which promotes a longer AP plateau [32,61]. Overall, Ca^2+^ release and reuptake are decreased in female CMs at baseline, and these differences can contribute to those seen in APD and contraction.

### 3.5. Male Myocytes Contract with Greater Velocity Compared to Females

**Figure 5:**
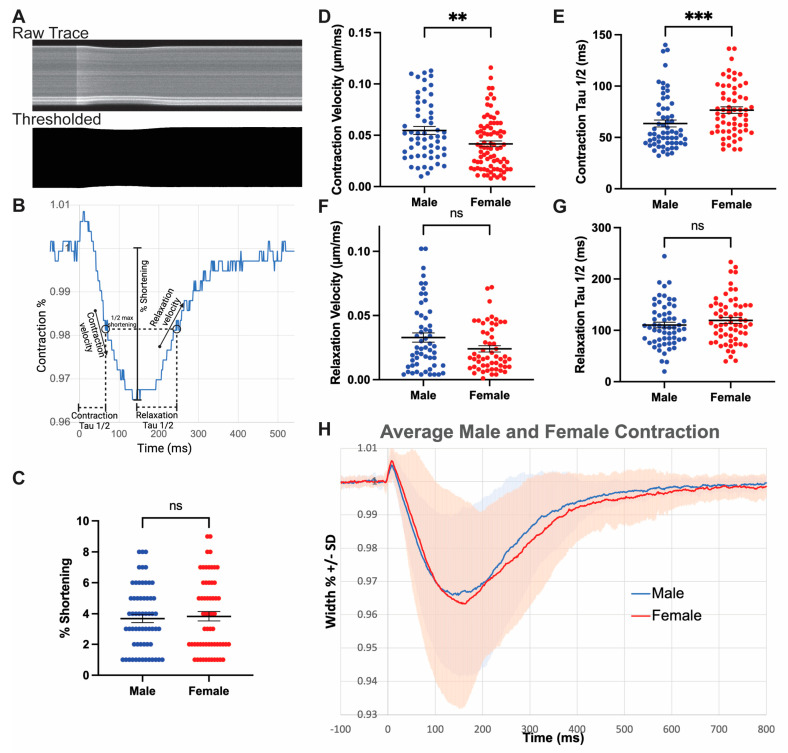
Increased contraction velocity and shortened contraction times in male myocytes. (**A**) Kymograph of raw voltage trace that is thresholded in ImageJ to allow quantification of contraction. (**B**) Representative contraction trace extracted from the thresholded kymograph. Key parameters are defined: contraction and relaxation velocity, % shortening from baseline, and 1/2 times for contraction and relaxation. Representative taken from a female cell with the following parameters: % shortening 3.4%, contraction velocity 0.037 μm/ms, contraction tau1/2 66.96 ms, relaxation velocity 0.013 μm/us, and Tau1/2 relaxation 110.36 ms. Scatterplots depicting (**C**) percent shortening, (**D**) ½ contraction time, (**E**) contraction velocity, (**F**) ½ relaxation time, and (**G**) relaxation velocity between males and females. (**H**) Composite graph depicting average contraction from males and females across all samples with standard deviation. Data presented from n = 5 mice, 84 male cells and 86 female cells. ns indicates *p* > 0.05, ** indicates *p* < 0.01, and *** indicates *p* < 0.001 Mann–Whitney test.

The release of Ca^2+^ into the cytoplasm activates contractile proteins [60]. Isolated cardiomyocytes contract along a longitudinal plane parallel to myosin filaments [63]. The sum of individual contractions in the heart generates force to increase pressure in the ventricles and eject blood through the arteries [63]. To quantify the contraction and relaxation of cardiomyocytes, we used data from the Fluo-volt imaging channel. Fluo-volt, despite being a voltage dye, permits high-resolution imaging of cell width as it concentrates on cell membranes. Thus, it has a higher fluorescent intensity near cell borders, allowing demarcation between the cell and the background media [64] (Figure 2). The raw voltage kymograph is thresholded using ImageJ to produce a binary image from which cell width is extracted (Figure 5A). Isolated cardiomyocyte contractions were analyzed for percent shortening along with contraction and relaxation velocity and duration (1/2 contraction/relaxation to peak) (Figure 5B). We found that males and females have similar overall shortening percentages (Figure 5C). Despite achieving the same percent shortening, female myocytes contract 19% more slowly than males (Figure 5D). This inversely correlates with the finding that male myocytes demonstrate a 29% increase in contraction velocity (Figure 5E). Maximum relaxation velocity is also increased in males by 31%, though no sex differences were observed in the time for half relaxation to occur (Figure 5F,G). These findings relate to the increased Ca^2+^ release and reuptake rates from the SR in males, which could stimulate faster rates of myofilament activation and increased contraction velocity, as well as faster release relaxation when Ca^2+^ is more rapidly removed from the cytosol. Overall, male myocytes contract and relax more quickly while demonstrating the same amount of total shortening (Figure 5H).

### 3.6. Sex Differences Exist in Coupling Times between Action Potential and Peak Contraction

**Figure 6:**
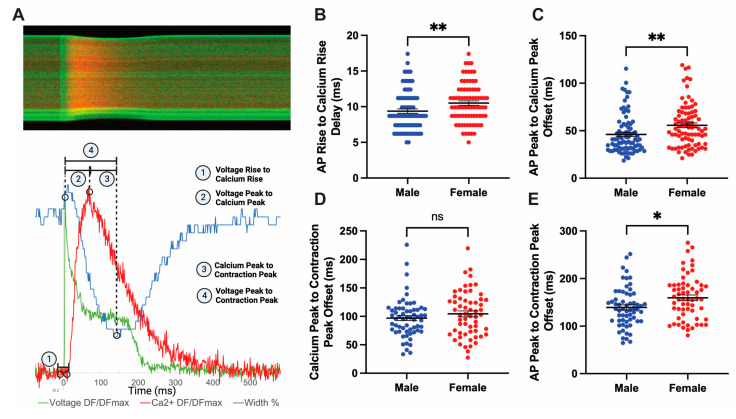
Sex differences exist in excitation-contraction coupling as increased times between peak action potential, Ca^2+^ concentration, and contraction in females. (**A**) Kymograph of membrane potential, Ca^2+^ transient, and contraction over 500 ms corresponding with representative composite trace extracted from the kymograph (green, action potential; red, Ca^2+^; blue, contraction) with key parameters identified. A representative from a female cell with the following parameters: AP rise to Ca^2+^ rise 13.54 ms, AP peak to Ca^2+^ peak 62 ms, Ca^2+^ peak to contraction peak 73.16 ms, AP peak to contraction peak 135.16 ms. Scatterplots depicting male and female differences in (**B**) AP rise to Ca^2+^ Rise, (**C**) AP peak to Ca^2+^ transient peak, (**D**) Ca^2+^ peak to contraction peak, and (**E**) voltage peak to contraction peak. Data presented from n = 5 mice, 84 male cells and 86 female cells. ns indicates *p* > 0.05, * indicates *p* < 0.05, and ** indicates *p* < 0.01 in Mann-Whitney test.

The coupling between membrane depolarization, Ca^2+^ release, and contraction is critically important in cardiac electrophysiology, but has previously been hard to determine due to rapid dynamics and the lack of an integrated detection method. Simultaneous recordings of AP, Ca^2+^, and contraction at 0.8 kHz resolution allow for precise determination of time intervals between these critical events (Figure 2). Simultaneous imaging generates a composite kymograph from which additional coupling parameters can be extracted and analyzed (Figure 6A). A short time separates AP rise from initial Ca^2+^ influx, near 10 ms in both males and females. Males exhibit a significant 1.1 ms average faster time from AP rise to calcium rise (Figure 6B). A greater time separates the AP peak from the Ca^2+^ peak as membrane depolarization occurs much more rapidly than Ca^2+^ release from the SR. We found that there were baseline sex differences in the time from the AP peak to the Ca^2+^ transient peak, occurring 19% faster in males than females (Figure 6C). The magnitude of this difference, 10 ms, was the same as the sex difference in Ca^2+^ transient rise time (Figure 4C). A similar finding was observed between peak Ca^2+^ transient and peak contraction as females were 7% slower on average (Figure 6D). This 8 ms difference corresponded with the difference in contraction ½ time between the sexes (Figure 5D). In congruence with these findings, the time from AP peak to contraction peak was also significantly shorter in male myocytes by 20 ms (Figure 6E). Overall, this indicates excitation-contraction coupling occurs more rapidly in males, with shorter average times from the arrival of an action potential to the peak of contraction. These studies offer a novel way to interpret the coupling between excitation and contraction and validate that sex differences exist in this basic physiological mechanism.

## 4. Discussion

Here, we present a method for the simultaneous detection of cardiac action potential, Ca^2+^ flux, and contraction while uncovering baseline sex differences in each event of excitation-contraction coupling. Our results demonstrate that baseline ventricular repolarization times, indicated by QTc and JTc intervals, were prolonged in C57Bl6/J female versus male mice at 4, 6, and 8 weeks of age. These findings were paralleled at the cellular level by the fact that female APD90 is increased compared with males in isolated CMs. Male CMs show increased rates of Ca^2+^ release and reuptake, suggesting baseline sex differences in SR Ca^2+^ handling. Additionally, male CMs exhibited increased contraction and relaxation velocities compared with females. The total time from CM depolarization to contraction was also shorter in males. In aggregate, these studies reveal baseline sex differences in cardiac physiology on both the organismal and cellular scale and suggest that sex differences in repolarization are widely conserved among species.

Our studies reveal baseline sex differences in the mouse QTc interval and APD. While these results mirror those observed in other mammals, there are conflicting results in C57Bl6/J mice [25,27,28,31]. We determined using power analysis that an n ≥ 14 of male and female mice would be necessary to establish significance at *p* < 0.05, assuming mice share a percent difference in QTc similar to humans. Therefore, the previous studies [25,27,28,31] of C57Bl6/J mice would likely not have had the statistical power to uncover sex differences. Although we did not control for menstrual cycle stage, the females in our study were likely not regularly cycling because they were housed independently and had not been exposed to male pheromones [65]. Thus, although hormone fluctuations are known to alter QTc, randomization of cycle stages should have controlled for these effects. Mice reach the onset of sexual maturity at 4–7 weeks. Our cohort of mice exhibited JTc and QTc differences at 4, 6, and 8 weeks. These findings indicate that sex differences in repolarization occur as early as 4 weeks of age, corresponding with the onset of sexual maturity. This relation corresponds with human data, which show that QTc interval differences are more prominent after puberty [66]. In sum, we observe greater female repolarization time in female mice at baseline, which indicates the C57 mouse model is suitable for determining the molecular mechanisms that account for differences in cardiac repolarization.

**Figure 7:**
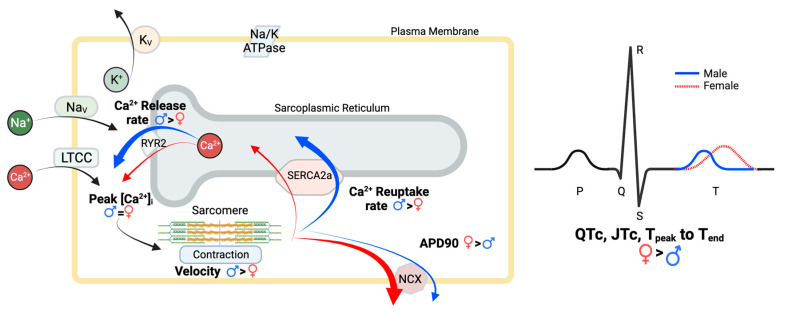
Overview of sex differences in excitation-contraction coupling. Female (red) and male (blue) differences in calcium handling and EKG are indicated with arrows.

We found that Ca^2+^ dynamics were altered between C57Bl6/J male and female mice. These results agree with studies by Parks et al. and Ceylan-Isik et al., who reported that Ca^2+^ release and reuptake rates are higher in male mice [33,34]. Longer times for Ca^2+^ reuptake in females correlate with prolonged female APD because Ca^2+^ handling has known impacts on action potential plateau. SERCA2a and the Na^+^-Ca^2+^ exchanger (NCX) are the primary contributors to intracellular calcium concentration decline in repolarization, though SERCA2a is more efficient [60]. Feedback through the electrogenic NCX can increase AP plateau in the cell if Ca^2+^ reuptake via SERCA2a is reduced, leading to increased repolarization times [61]. Previous reports have implicated greater NCX expression in female rats as well as greater SERCA2a expression in male mice [34,46,67]. Together, these expression differences could contribute to many of the observed excitation-contraction coupling differences (Figure 7). Therefore, modulating the rate of Ca^2+^ reuptake into the SR could also contribute to QTc differences in males and females. These male and female differences in Ca^2+^ handling are attributed to sex differences in cAMP and IGF-1 levels that impact the activity of calcium-handling proteins and SERCA2a expression, respectively [33,34]. Sex differences in Ca^2+^ handling are important to consider, because they could alter the propensity towards developing both arrhythmias and Ca^2+^ overload pathologies.

Contraction differences likely occur due to the sex differences in Ca^2+^ handling. Higher rates of Ca^2+^ release in males can speed the kinetics of Ca^2+^ binding to Troponin C and subsequent activation of cross-bridge formation [60]. This could correspond to greater contraction velocities because more actin–myosin complexes are engaged simultaneously, whereas longer times for Ca^2+^ release would slow kinetics and velocity. Alternatively, sex differences could also be explained by differences in myosin ATPase activity or alterations in sarcomere protein expression. Similarly, greater rates of Ca^2+^ reuptake would speed the kinetics of Ca^2+^ detachment from Troponin C, enabling more rapid relaxation rates [68]. Our results are in accordance with other studies that show greater contraction and relaxation velocities in males [32,34,36,37,38]. We did not observe differences in peak shortening, although this absence could be due to differences in mouse strain or age.

The imaging technique presented here improves upon previous methods to investigate CM physiology as it is minimally technical, highly processive, and detects triad parameters. The Lagendorff-free CM isolation technique used here, in combination with membrane-permeable fluorescent dyes and a relatively straightforward microscope setup, makes this procedure accessible to labs that may not have standard electrophysiology equipment. Although this method does not give absolute values of current, results can be obtained from many individual cells quickly as the imaging methodology does not require the technical challenge of patch clamping. The 0.8 kHz temporal resolution achieved with this technique offers a greater than two-fold improvement over previous methods in stem-cell-derived CMs [43]. Other previous techniques are not practical for isolated cardiomyocytes due to the necessity of expressing fluorescent proteins and the use of custom hardware [44]. Additionally, the analysis pipeline provided in the supplementary materials only requires the use of FIJI and Microsoft Excel, two easily accessible platforms that are typically more familiar to researchers than more specialized software. Coupling detection of the action potential, Ca^2+^ transient, and contraction serves as the best indicator of cell physiology and enables maximal physiological data acquisition from a single experiment. Our studies of coupling revealed longer times in females from peak depolarization to peak contraction. This difference was underpinned by the increased time from peak depolarization to peak calcium release in females. These results imply that sex differences in calcium rise time are responsible for differences in the overall duration of excitation-contraction coupling. Quantification of coupling between all the excitation-contraction events has not previously been investigated. Therefore, this study offers novel insight into the temporal dynamics of isolated CMs. These data are critical as it establishes a baseline for male and female coupling that can be compared to changes that may occur in a disease context. In addition, the results herein support the use of mouse models as surrogates for human arrhythmias, as the key human sex differences that influence arrhythmia prevalence are paralleled in our studies.

Molecular sex differences can act as a predisposition for acquired arrhythmias. Baseline differences in repolarization highlight this fact as males more often develop early re-entry arrhythmias like atrial fibrillation and short QT syndrome, while females are more prone to late repolarization arrhythmias like Long QT syndrome and torsades de pointes [8,9,10,11,69]. Sex differences are established by both hormonal and sex chromosome dosage effects that alter the cellular environment from the epigenomic to the post-translational level [46]. Sex differences in repolarization are attributed to hormonal influence. However, differential protein expression controlled by another mechanism, like chromosome dosage, could also contribute to the sex differences in repolarization. Due to off-target effects, hormonal therapies are not ideal for the treatment of arrhythmias. Altering specific protein expression or activity could offer a better avenue for targeted therapies.

Our study provides a novel tool to simultaneously record and interpret the physiological parameters of excitation-contraction coupling in isolated CMs. Recording kinetics at room temperature was a major limitation of these studies, as physiological temperatures could yield different results. Many of the previous studies referenced have uncovered similar sex differences at 37 °C [26,33,34]. While temperature control was not an option given our current hardware, we plan to implement it in future experiments. Although these studies were limited to wild-type C57Bl6/J male and female mice, investigators could adapt this system for genetically modified mouse strains or drug models of cardiac disease that would aid in uncovering the molecular dynamics that alter excitation-contraction coupling. It will be critical to determine the proteins and pathways that can serve as druggable targets to treat sex-biased cardiac arrhythmias. While previous research has largely focused on hormonal influences, it may prove fruitful to investigate sex differences that can be accounted for by sex chromosome dosage. Overall, a better understanding of baseline sex differences in electrophysiology can offer new and improved avenues for treating sex-biased cardiac diseases.

## Data Availability

All protocols and data from this study will be made available upon request to the senior author. All macros, data analysis Excel sheets, and sample data sets are available in the supplementary materials.

## Supplementary Materials

All ImageJ macros, Excel analysis sheets, and an example dataset can be downloaded at: https://cdr.lib.unc.edu/collections/k3569f699 (accessed on 11 November 2023). Supplementary figures can be downloaded at https://www.mdpi.com/article/10.3390/jcdd10120479/s1. Figure S1: Power analysis to determine EKG sample size; Figure S2: EKG parameters at 4, 6, and 8 weeks; Table S1: CM isolation buffer compositions; Figure S3: Sex differences in excitation contraction coupling.
