# Supplementary material for: Sex Differences in Mouse Cardiac Electrophysiology Revealed by Simultaneous Imaging of Excitation-Contraction Coupling
Source: J Cardiovasc Dev Dis. 2023 Nov 29;10(12):479. doi: 10.3390/jcdd10120479 (PMC10743987; doi:10.3390/jcdd10120479)
Supplement: Supplementary file 1 [file jcdd-10-00479-s001.zip › methods buffers.pdf]

CM isolation buffer compositions:

EDTA Buffer:

Compound	Molar mass (g/mol)	Final concentration (mmol/l)	g/500ml required
NaCl	58.44	130	3.8
KCl	74.55	5	0.185
$Na_2HPO_4.7H_2O$	268.07	0.5	0.076
HEPES	238.3	10	1.19
Glucose (Dextrose)	180.16	10	0.9
BDM	101.1	10	0.5
Taruine	125.15	10	0.625
EDTA.2Na.$2H_20$	372.24	5	0.73

Adjust pH to 7.8 with NaOH sterile 0.22µm filter and store at 4˚ C

Perfusion Buffer:

Compound	Molar mass (g/mol)	Final concentration (mmol/l)	g/500ml required
NaCl	58.44	130	3.8
KCl	74.55	5	0.185
$Na_2HPO_4.7H_2O$	268.07	0.5	0.076
HEPES	238.3	10	1.19
Glucose (Dextrose)	180.16	10	0.9
BDM	101.1	10	0.5
Taruine	125.15	10	0.625
$MgCl_2.6H_2O$	203.3	1	0.101

Adjust pH to 7.8 with NaOH sterile 0.22µm filter and store at 4˚ C

Collagenase Buffer:

Compound	Final concentration	Concentration of stock
Collagenase II	0.5 (mg/ml)	100x (50mg/ml)
Collagenase IV	0.5 (mg/ml)	100x (50mg/ml)
Protease XIV	0.05 (mg/ml)	1000x (50mg/ml)
$CaCl_2$	50 µM	2000x (100mM)

Make collagenase buffer day of experiment

STOP Buffer:

Solution	ml for 10ml STOP buffer
Perfusion buffer	9.5
FBS	0.5

Culture/ Imaging media:

Compound	Stock concentration	Final concentration	ml / 100ml media required
M199 Media (no phenol red)	/	/	95
BSA	5% (in PBS)	0.1%	2
ITS	100x	1x	1
CD lipid	100x	1x	1
Penstrep	100x	1x	1

Store Culture media at 4˚C

Calcium Reintroduction Buffer:

Solution	ml for Buffer I	ml for Buffer II	ml for Buffer III
Perfusion buffer	15	10	5
Culture media	5	10	15

Store Calcium reintroduction buffers at 4˚C

Critical Reagents source:

Reagent	Vendor	Cat #
Collagenase type II powder	Thermofisher	17101015
Collagenase type IV powder	Thermofisher	17104019
Protease type XIV powder	Sigma	P5147-100MG
M199 media (no phenol red)	Thermofisher	11043023
ITS supplement	Sigma	I3146-5ML
CD lipid concentrate	Thermofisher	11905031
