# Supplementary material for: Sex Differences in Mouse Cardiac Electrophysiology Revealed by Simultaneous Imaging of Excitation-Contraction Coupling
Source: J Cardiovasc Dev Dis. 2023 Nov 29;10(12):479. doi: 10.3390/jcdd10120479 (PMC10743987; doi:10.3390/jcdd10120479)
Supplement: Supplementary file 1 [file jcdd-10-00479-s001.zip › Supplmental figures JCDD Emerson.pdf]

Supplementary Figures:

$$k = \frac{n_2}{n_1} = 1$$

$$n_1 = \frac{(\sigma_1^2 + \sigma_2^2/K)(z_{1-\alpha/2} + z_{1-\beta})^2}{\Delta^2}$$

$$n_1 = \frac{(19^2 + 19^2/1)(1.96 + 0.84)^2}{20^2}$$

$$n_1 = 14$$

$$n_2 = K * n_1 = 14$$

Δ = |μ2-μ1| = absolute difference between two means

σ1, σ2 = variance of mean #1 (male) and #2 (female)

n1 = sample size for group #1 (female)

n2 = sample size for group #2 (male)

α = probability of type I error (0.05)

β = probability of type II error (0.2)

z = critical Z value for a given α or β

k = ratio of sample size for group #2 to group #1

Supplemental Figure s1: Power analysis to determine sample size for electrocardiogram (EKG) experiments. Given that the standard QT interval difference in males and females is 20 ms and assuming a minimal standard deviation of 19ms, the following power analysis formula was used to determine the minimal sample size needed to determine if C57Bl/6J mice recapitulate human differences in EKG recordings [27-32].

	4 weeks		6 weeks		8 weeks	
	**male n=14**	**female n=15**	**male n=15**	**female n=17**	**male n=16**	**female n=14**
RR Interval (ms)	138.4 ± 10.9	120.6 ± 8.07	109.8 ± 5.14	121.1 ± 6.13	113.7 ± 5.72	112.6 ± 7.02
Heart Rate (BPM)	478.0 ± 34.9	539.3 ± 32.5	568.2 ± 25.3	529.0 ± 27.4	549.9 ± 25.8	567.5 ± 30.6
PR Interval (ms)	30.15 ± 0.65 [ab]	30.57 ± 0.32	33.52 ± 1.04 [a]	31.68 ± 0.44	34.05 ± 0.66 [b]	32.44 ± 0.64
P Duration (ms)	11.88 ± 0.81	12.13 ± 0.71	12.74 ± 0.93	11.50 ± 0.42	12.57 ± 0.98	12.28 ± 0.77
QRS Interval (ms)	8.50 ± 0.22	8.311 ± 0.29 [ab]	8.733 ± 0.36	9.579 ± 0.37 [a]	9.420 ± 0.25	9.764 ± 0.29 [b]
QT Interval (ms)	15.97 ± 0.56	17.79 ± 0.52	13.82 ± 0.73	17.88 ± 1.01	15.08 ± 0.40	17.27 ± 0.83
QTc (ms)	44.93 ± 2.48	53.58 ± 2.96	42.39 ± 2.52	53.37 ± 3.25	45.78 ± 2.12	53.77 ± 2.98
JT Interval (ms)	7.390 ± 0.55	9.488 ± 0.57	4.961 ± 0.53	8.309 ± 0.92	5.430 ± 0.48	7.497 ± 0.72
JTc (ms)	36.42 ± 2.38	45.27 ± 2.99	33.65 ± 2.33	43.79 ± 3.08	36.36 ± 2.04	44.08 ± 2.89
Tpeak Tend Interval (ms)	4.832 ± 0.48	6.565 ± 0.43	2.752 ± 0.34	5.307 ± 0.78	3.427 ± 0.40	5.052 ± 0.52
P Amplitude (mV)	64.48 ± 11.0	84.45 ± 11.6	19.28 ± 13.8	33.62 ± 14.5	20.33 ± 14.1	45.24 ± 14.4
Q Amplitude (mV)	18.88 ± 4.53	25.99 ± 6.00	8.272 ± 3.61	8.466 ± 8.14	4.974 ± 3.35	5.428 ± 6.30
R Amplitude (mV)	863.9 ± 88.6	933.9 ± 89.9	652.6 ± 83.8	727.3 ± 55.8	582.6 ± 57.0	658.1 ± 51.2
S Amplitude (mV)	-340.2 ± 71.6	-198.2 ± 35.1	-333.1 ± 72.7	-258.5 ± 47.4	-432.2 ± 60.9	-264.8 ± 45.8
ST Height (mV)	38.58 ± 8.44	74.12 ± 9.13 [ab]	16.05 ± 10.0	26.06 ± 13.7 [a]	14.86 ± 10.6	20.19 ± 14.3 [b]
T Amplitude (mV)	107.5 ± 11.9	149.9 ± 16.1	97.10 ± 14.7	116.8 ± 13.9	133.6 ± 16.4	106.7 ± 14.4

Supplemental Figure S2: EKG recordings of mice aged 4, 6, and 8 weeks. Data was analyzed using ANOVA with Tukey's multiple comparisons test for differences in sex and age. Significant differences $p<0.05$ are indicated by darker shading when sex differences occur between males and females at the same time point, or by corresponding letters when change occurs in the same sex over different time points. Data is represented as mean ± standard error of the mean.

Sex Differences in Excitation-Contraction Coupling	Male	Female	P-value
**Calcium Handling**			
DF/F0	1.17 ± 0.07	1.17 ± 0.08	0.9442
Rise time (ms)	40.4 ± 2.0	47.3 ± 2.1	0.0181
Tau 1/2 (ms)	87.8 ± 2.3	101.2 ± 2.7	0.0002
Tau 90 (ms)	213.1 ± 4.3	234.1 ± 5.4	0.0026
**Voltage**			
DF/F0	0.276 ± 0.006	0.294 ± 0.006	0.0314
Rise time (ms)	4.43 ± 0.12	4.75 ± 0.16	0.1015
Tau 90 (ms)	101.0 ± 10.2	146.9 ± 14.4	0.0097
**Ca2+ vs Voltage**			
AP Delay to Ca2+ rise start (ms)	9.36 ± 0.31	10.50 ± 0.31	0.0101
AP to Ca2+ Peak offset (ms)	46.1 ± 2.2	55.7 ± 2.5	0.0047
**Width**			
% change in width	3.68 ± 0.27	3.83 ± 0.31	0.7217
Contraction time (ms)	144.1 ± 5.5	164.2 ± 6.0	0.0152
Tau 1/2 relaxation (ms)	110.3 ± 5.6	119.3 ± 5.8	0.2646
Tau 1/2 contraction (ms)	63.6 ± 3.4	76.6 ± 3.2	0.0059
Contraction velocity (um/ms)	0.055 ± 0.004	0.041 ± 0.003	0.0077
Relaxation velocity (um/ms)	0.033 ± 0.004	0.024 ± 0.002	0.0437
**Width vs Ca2+**			
Ca2+ peak to contraction offset (ms)	96.9 ± 4.4	104.4 ± 5.2	0.2711
AP peak to contraction min offset (ms)	139.5 ± 5.4	159.6 ± 6.0	0.0130

Supplemental Figure S3: Overview of all excitation-contraction coupling differences between male and female isolated cardiomyocytes from n=5 mice, 84 male, and 86 female cells. P-values were determined by students' two-sample t-test and are indicated in the rightmost column. Data is represented as mean ± standard error of the mean.
